# Effect of Weather Conditions on Phytochemical Profiles in Organically Grown Cowpea (*Vigna unguiculata* L. Walp)

**DOI:** 10.3390/plants14203179

**Published:** 2025-10-16

**Authors:** Jamila M. Mweta, Getrude G. Kanyairita, Franklin Quarcoo, Faraja Makwinja, Daniel A. Abugri, Gregory Bernard, Toufic Nashar, Desmond G. Mortley, Melissa Boersma, Conrad K. Bonsi

**Affiliations:** 1 Department of Agricultural and Environmental Sciences, College of Agriculture, Environment and Nutrition Sciences, Tuskegee University, Tuskegee, AL 36088, USA; gkanyairita5762@tuskegee.edu (G.G.K.); fquarcoo1@tuskegee.edu (F.Q.); gbernard@tuskegee.edu (G.B.); dmortley@tuskegee.edu (D.G.M.); 2 Department of Crop Science & Beekeeping Technology, College of Agricultural Sciences and Food Technology, University of Dar es Salaam, Dar es Salaam P.O. Box 35091, Tanzania; makwinja.faraja@udsm.ac.tz; 3 Department of Biological Sciences, Microbiology PhD Program, College of Science, Technology, Engineering and Mathematics, Alabama State University, Montgomery, AL 36104, USA; dabugri@alasu.edu; 4 Department of Pathobiology, College of Veterinary Medicine, Tuskegee University, Tuskegee, AL 36088, USA; tnashar@tuskegee.edu; 5 Department of Chemistry and Biochemistry, Mass Spectrometry Center, College of Science and Mathematics, Auburn University, Auburn, AL 36849, USA; mdb0067@auburn.edu

**Keywords:** cowpea varieties, seasons, phytochemicals, chromatography-mass spectrometry, liquid chromatography-tandem mass spectrometry

## Abstract

Cowpeas are prone to abiotic (heat and drought) and biotic (pathogens and insect pests) stresses, with the former representing the predominant challenge, causing poor growth and reduced yield globally under changing climatic conditions. Cowpea can synthesize phytochemicals to respond to these stresses; however, there is limited information on the impact of weather on phytochemical biosynthesis in the cowpea phyllosphere. Phytochemical profiles were determined via chromatographic and spectrophotometric analyses of leaf samples from six cowpea varieties grown during 2020–2021. A total of 10 fatty acid methyl esters (FAMEs) and 62 diverse metabolites were identified across varieties and seasons, with higher levels in 2020 under elevated temperatures and rainfall. The Queen Anne (QA) variety exhibited the maximum concentration of elaidic oleic acid (cis + trans), behenate, lignocerate, methyl laurate, and methyl palmitate (with the highest concentration at 258.415 µg/mL), and the Whippoorwill Steele’s Black (WP) variety predominantly exhibited diverse phytochemicals with high peak areas during 2020, including phenolic acids, phytohormones, alkaloids, flavonoids, and amino acids. While higher overall increases were observed in 2020, some compounds and varieties peaked in 2021, including FAMEs in the Colossus (CL) variety and other phytochemicals in QA. Flavonoid, flavone, and flavonol biosynthesis; phenylalanine metabolism; and tyrosine metabolism were significantly affected, leading to the accumulation of metabolites. Understanding plant–climate interactions will help farmers with variety selection and planting decisions. This study suggests that further research on the temperature mechanism for the biosynthetic pathways of these metabolites in the screened cowpea varieties is required.

## 1. Introduction

Cowpea is an important commercial crop and a highly nutritious vegetable due to its rich nutrient composition, consisting of proteins, carbohydrates, minerals, and vitamins. It contributes significantly to the global economy through its diverse applications as a grain crop, livestock feed, and vegetable [1,2,3,4,5,6,7]. This crop is grown in several countries around the world [7,8] and serves as a cover crop, effectively suppressing weeds, soilborne diseases, and nematodes, while also providing organic matter, improving soil structure, and reducing soil erosion. Furthermore, cowpea can fix nitrogen through a symbiotic relationship with microorganisms in the rhizosphere, making it a cost-effective and sustainable alternative to inorganic fertilizers [9,10]. However, cowpea production is impeded by abiotic (including environmental factors such as heat, reactive oxygen species, cold, drought, and salt) and biotic (pathogens and insect pests) stresses throughout its life cycle, from seed storage to seedling development and maturity [11,12,13,14]. Abiotic stresses are considered the most challenging stressors in crop production, affecting plant growth and yield worldwide [14]. The cowpea phyllosphere has evolved defense mechanisms against these abiotic and biotic stresses, including the synthesis of phytochemicals (plant metabolites), which function as defenders against extreme weather, antimicrobial agents, and insecticides [15,16,17].

In recent years, increasing consumer concerns about food safety and quality, soil conservation, and environmental safety have led to an increase in the adoption of sustainable agriculture practices [18]. Organic agriculture, a fundamental pillar of sustainable agriculture, employs ecologically friendly pest control techniques [19] such as safe biopesticides, crop rotation, and sanitation. Plant-based pesticides are central to this approach, functioning as signaling molecules with antimicrobial, insecticidal, and antioxidant activities that enhance plant resilience to both biotic and abiotic stresses. These compounds include volatile and non-volatile phytochemicals such as terpenoids; fatty acids and fatty acid derivatives; phytohormones such as salicylic acid, indole acetic acid, abscisic acid, and gibberellic acid; and phenolics, including phenolic acids and flavonoids [14,15,20,21,22,23]. Non-volatile phytochemicals contribute significantly to stress adaptation. For example, flavonoids accumulate in the roots in response to salinity [24], and quercetin accumulates under drought stress [25]. Volatile phytochemicals such as terpenes and fatty acids also play a critical role: saturated fatty acids (such as palmitic, myristic, and stearic acids) [26,27] and unsaturated fatty acids (UFAs), especially compounds with 18-carbon-long UFAs like oleic (18:1n9), alpha-linolenic (18:3n3), and linoleic (18:2n6) acids, are mobilized during stress and subsequently converted into bioactive molecules [28], including azelaic, jasmonic, and 2-hydroxy-octadecatrienoic acids via oxidation [16]. Fatty acids have multifaceted roles in plant health. They act as repellents, signaling molecules that activate the defense mechanism of the plant, sources of biosynthesis of other bioactive phytochemicals, and reservoirs of components for the synthesis of the extracellular barriers of the plant [16,29,30]. Similarly, terpenes strengthen defense mechanisms by acting as attractants for natural enemies, insect repellents, and pathogen growth inhibitors, as well as reducing oxidative stress [31,32,33,34,35,36].

The biosynthesis of phytochemicals in plants can be affected by weather conditions such as variations in rainfall and temperature, enabling them to modify their phytochemical profiles in response to stress via phenotypic plasticity [37]. For example, Gitonga et al. [38] found that environmental conditions affect the synthesis of phytochemicals in cowpea, with phytochemical contents varying significantly between environments, including increased production in the long rainy season. Mansour-Gueddes et al. [39] reported a significant increase in the contents of phenolic compounds, tannins, saponins, flavonoids, phytosterols, and carotenoids, along with a decrease in chlorophyll levels, in olive tree leaves grown in a lower arid (southern) area compared to the subhumid (northern) and higher semi-arid (central) areas of Tunisia. Higher antioxidant activity contributes to a reduction in oxidative stress in olive trees, suggesting that the increase in the phytochemicals observed in the study can be interpreted as the tree’s reaction to environmental threats and oxidative stress caused by the harsh weather typical of the southern area. High temperatures and rainfall can increase pest populations and exacerbate disease due to excessive moisture, which creates favorable conditions for pathogens [40], triggering an increase in the synthesis of defensive compounds. Insects cause direct plant damage, with some, such as aphids, serving as vectors for plant pathogens, such as viruses [41], which can further influence metabolite production.

Extremely high temperatures are considered the most challenging weather conditions, causing heat stress in plants that, in turn, causes them to initiate defensive mechanisms by redirecting carbon from growth and reproduction (primary metabolism) to bioactive compound biosynthesis (secondary metabolism) [42]. High temperatures regulate gene expression, enzyme activities, and plant hormones, affecting alkaloid and volatile terpene biosynthesis. They can also cause reactive oxygen species to form, which in turn activate the biosynthesis of antioxidant secondary metabolites such as phenolics and flavonoids in plants [43,44].

Field-based cowpea research is important for providing essential insights into climate-adaptive agriculture in uncontrolled environments that greenhouse studies are limited to. Agricultural systems are dependent on climate stability; changing climatic conditions cause various challenges that impact agricultural activities through intensified biotic and abiotic stressors [45]. Natural field conditions expose cowpeas to complex environmental stressors, including fluctuations in temperature, light, rainfall, and soil conditions that elicit adaptation responses. The selection of varieties with better adaptive traits and important weather information is essential. Other studies show that farmers who used weather and climate information services and better selection of the cowpea varieties obtained significantly higher yields, with 34% higher gross margins than those who did not [46]. Stressful field conditions may induce greater facilitation and more positive plant–soil feedback [47], revealing a metabolic flexibility that is essential for developing climate-resilient varieties capable of maintaining productivity under increasingly variable environmental conditions.

Fluctuations in phytocompounds in plants affect their defense mechanisms against abiotic and biotic stresses. Although there are studies on the effects of weather in cowpea production, few studies link weather information and phytocompound expression with primary functions such as defense against biotic and abiotic stresses. Therefore, the necessity arises to determine whether the weather fluctuations in Alabama during the summer can impact the phytochemical composition of the cowpeas. This study aims to investigate the effect of seasonal variations on the composition and concentration of the phytochemical compounds produced by cowpeas. This research will assist farmers around the world in selecting optimal cowpea varieties that produce essential phytochemicals to defend against changing weather conditions.

## 2. Results

### 2.1. Weather Data

Statistical analysis of weather data for the two seasons revealed significant differences in climatic parameters between 2020 and 2021, including temperature and rainfall, where the difference was highly significant between the two seasons (*p* < 0.0001). In contrast, there were no significant differences in relative humidity between the 2020 and 2021 growing seasons (*p* = 0.74). These findings indicate that temperature and rainfall were the most substantially different climatic factors between the two study periods, while humidity remained relatively consistent in both seasons (Table 1).

The average temperature for seasons and individual months within the season is shown in Table 1. In 2020, the temperature (32.02 °C) was higher than in 2021 (29.88 °C), indicating that 2020 was the warmer of the two years. In 2020, July and August were the hottest months, with temperatures of approximately 33 °C, and were slightly warmer than June, averaging 31 °C. In 2021, August was the hottest (31 °C), compared to June and July, for which the average temperatures were 29.5 °C and 28.8 °C, respectively. Weather data show that 2020 was significantly warmer than 2021 in all months; therefore, cowpeas in this year were exposed to prolonged heat stress (consistent maximum temperature of 32.02 °C). The highly significant *p*-values (<0.0001) and the small standard errors show that the results of environmental difference analysis between 2020 and 2021 are robust, not random variations.

### 2.2. Identification of Targeted Phytochemicals Using GC-MS

**Figure 1:**
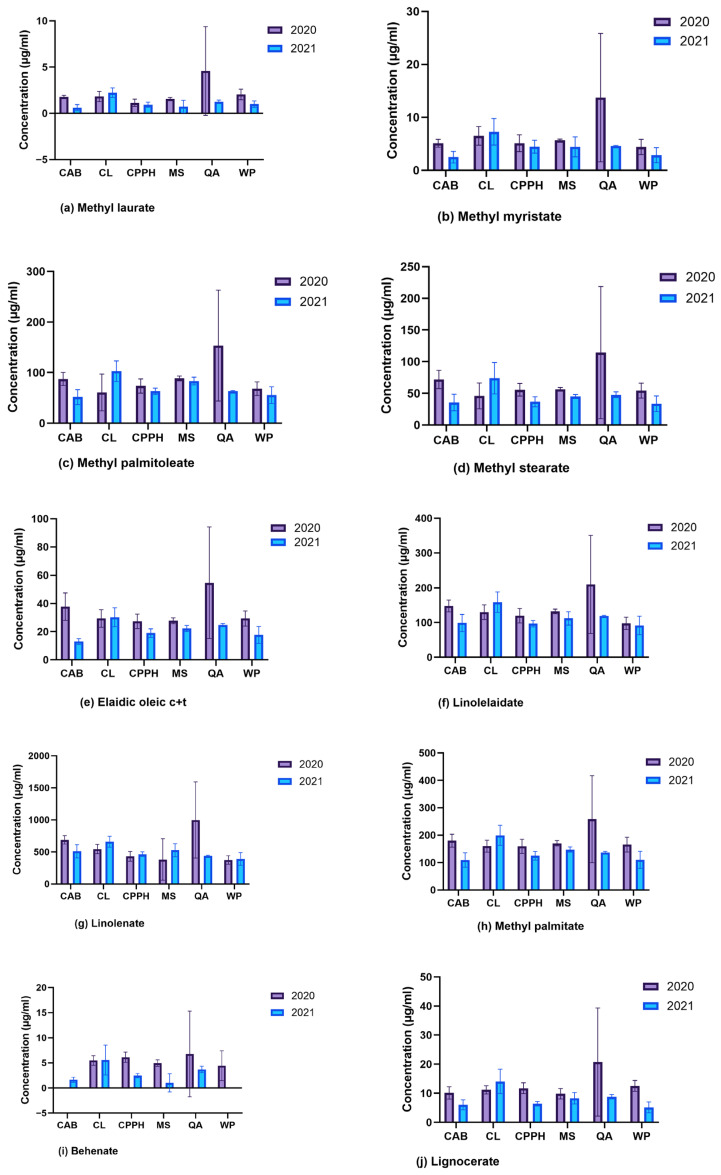
Mean concentrations of FAMEs identified between cowpea varieties and seasons (2020 and 2021), with their names indicated (**a**–**j**). Note: CPPH—Coronet Pinkeye Purple Hull, CL—Colossus, CAB—California Blackeye Pea, WP—Whippoorwill Steele’s Black, QA—Queen Anne, and MS—Mississippi Silver. (**a**) Methyl laurate, (**b**) methyl myristate, (**c**) methyl palmitoleate, (**d**) methyl stearate, (**e**) elaidic oleic c + t, (**f**) linolelaidate, (**g**) linolenate, (**h**) methyl palmitate, (**i**) behenate, and (**j**) lignocerate.

Analysis of the fatty acid composition revealed that ten fatty acid methyl esters were detected at significant concentrations, as shown in Figure 1a–j: methyl stearate (18:0), methyl palmitoleate (16:1), methyl palmitate (16:0), methyl myristate (14:1), methyl laurate (12:0), elaidic oleic acid (cis + trans; 18:1) or elaidic oleic c + t (18:1), lignocerate (24:0), behenate (22:0), linolenate (a methyl ester of linolenic acid; 18:3n3), and linolelaidate (methyl linolelaidate or methyl trans,trans-9,12-octadecadienoate; 18:2n6). The other two compounds, methyl decanoate (10:0) and linolenate (18:3n6), were not detected in any cowpea variety; therefore, they were excluded from the study. Methyl stearate, methyl palmitoleate, methyl palmitate, methyl myristate, and methyl laurate are the methyl esters of the following fatty acids: stearic acid, palmitoleic acid, palmitic acid, myristic acid, and lauric acid, respectively. The fatty acid composition varies by cowpea variety and season, as evidenced by the identified FAMEs.

Analysis of variance (ANOVA), followed by pairwise comparisons using Tukey’s post hoc test (raw *p* < 0.05), was conducted to identify significant differences in mean compound concentrations among varieties and growing seasons. The amount of methyl laurate (*p* = 0.04), methyl palmitate (*p* = 0.02), elaidic oleic c + t (*p* = 0.003), behenate (*p* = 0.02), and lignocerate (*p* = 0.02) varied significantly between seasons, with the highest concentration of these compounds observed in samples from 2020. QA exhibited the highest concentrations of all compounds detected in 2020, and CL exhibited the highest concentrations of all compounds in 2021, while the low concentrations of most compounds were detected in CAB, WP, CPPH, and MS (except for methyl palmitoleate and methyl stearate, in both seasons). Methyl palmitate was highly abundant in the QA variety (258.415 µg/mL) compared to other significant FAMEs in all varieties. Lignocerate was highest in the QA variety (2020), followed by the CL (2021), and lowest in the CAB and WP varieties in 2021. Behenate was not detected in the CAB and the WP varieties in 2020 and 2021, respectively. Methyl laurate and behenate (1 μg/mL) were the least abundant in the CAB and MS varieties, respectively, in 2021. In 2021, the CAB variety had the lowest concentration of many compounds, including elaidic oleic c + t, methyl laurate, and methyl myristate (Figure 1a–j).

### 2.3. Identification of Untargeted Phytochemicals with LC-MS/MS

In the LC-MS/MS analysis, out of 68 tentatively matched metabolites, 62 were significantly different, including volatile phytochemicals such as terpenoids (oleanolic (or ursolic) acid and carvone); fatty acids and their derivatives (linoleic acid, alpha-linolenic acid, palmitic acid, azelaic acid, suberic acid, corchorifatty acid F, palmitic amide, 16-hydroxyhexadecanoic acid, linoleoyl ethanolamide, oleamide, and octadecanamide); phytohormones (salicylic acid and jasmonic acid); non-volatile phytochemicals including flavonoids (kaempferol, catechol, and isoliquiritigenin), alkaloids (vindoline, reserpine, and isoquinoline), polyphenolic pigments (pelargonidin), and phenolic acids (homogentisic acid, gentisic acid, ferulic acid, trans-cinnamic acid, and gallic acid); and primary metabolites like amino acids (phenylalanine, isoleucine, leucine, and proline).

Two-way ANOVA was performed, and an adjusted *p*-value (FDR < 0.05) was used as the significance threshold to ensure robust findings. The number of phytochemicals that were significantly affected by season, cowpea variety, and their interactions is shown in Table A1. Of the 68 phytochemicals, 62 were significantly affected by these factors (FDR < 0.05), with the unique phytochemicals affected by each factor and their combined effects. Three tentatively identified metabolites, kaempferol (*p* = 0.02), adenosine (*p* = 0.02), and phenylacetaldehyde (*p* = 0.03), showed significant differences between varieties regardless of the season, and uracil, proline, jasmonic acid, and cuminaldehyde varied significantly between seasons, reflecting that neither their production nor accumulation was dependent on variety. Alpha-linolenoyl ethanolamide was influenced by the interaction between season and variety; neither the variety nor the seasonal change alone had a significant effect (Table A1).

The shared significance is shown by the overlap effects of the phytochemicals (FDR < 0.05) (Table A1), whereas argininosuccinic acid, linoleoyl ethanolamide, 2-piperidinone, and alpha-linolenic acid were significantly affected by variety and season. Both the cowpea variety and the season independently influenced the levels of these phytochemicals. Isoliquiritigenin, apigenin, kynurenic acid, carvone, isoquinoline, and ergocalciferol were strongly influenced by cowpea variety and the interaction between cowpea variety and season. Neocnidilide, palmitic amide, corchorifatty acid F, dihydrotestosterone, reserpine, leucylproline, octadecanamide, and 4-hydroxyphenylpyruvic acid were influenced by both the season and interactions between season and variety. The remaining 36 phytochemicals were significantly affected by all three factors (cowpea variety, season, and interaction), demonstrating a strong combined effect (FDR < 0.05) and thus indicating robust results beyond random chance. These phytochemicals are glycitein, rhamnetin, aesculin, D-malic acid, phenol, salicylic acid, chrysin, ferulic acid, homogentisic acid, gentisic acid, trans-cinnamic acid, catechol, daidzin, pelargonidin, gallic acid, L-phenylalanine, vindoline, benzoic acid, 3-cresotinic acid (3-methylsalicylic acid), (Z)-3-methyl-2-(2-pentenyl)-2-cyclopenten-1-one, xanthine, 16-hydroxy hexadecanoic acid, 4-hydroxybenzaldehyde, oleamide, ortho-hydroxyphenylacetic acid, isoleucine, oleanolic acid, linoleic acid, suberic acid, fumaric acid, azelaic acid, guanine, palmitic acid, ethyl tetradecanoate, leucine, and thymine (Table A1).

**Figure 2:**
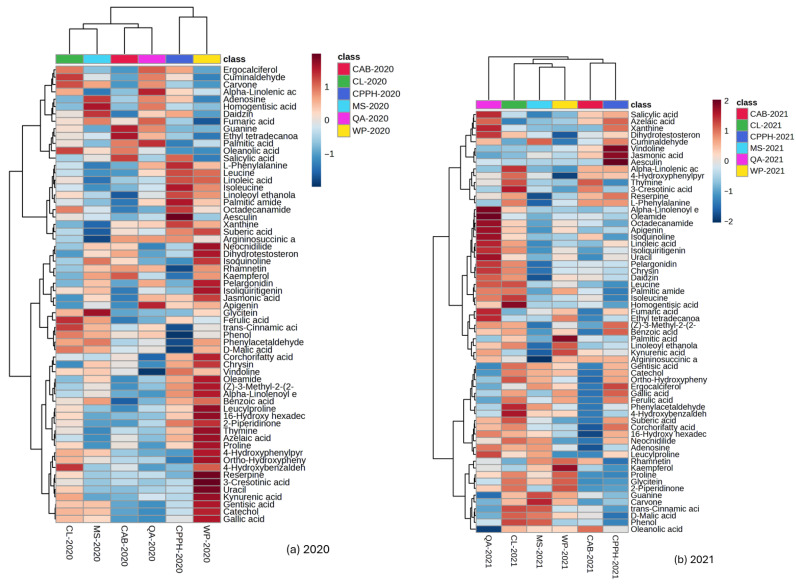
Heatmap visualization of significantly affected phytochemicals in six cowpea varieties across two growing seasons: (**a**) 2020 and (**b**) 2021. Color intensity represents relative peak area abundance, with red indicating high metabolite levels and blue indicating low metabolite levels. Each row represents a distinct phytochemical compound, and each column represents a cowpea variety. The heatmap illustrates seasonal variation in phytochemical profiles and variety-specific metabolic responses across the experimental period. CPPH—Coronet Pinkeye Purple Hull, CL—Colossus, CAB—California Blackeye Pea, WP—Whippoorwill Steele’s Black, QA—Queen Anne, and MS—Mississippi Silver.

A hierarchical heatmap was used to visualize metabolic profiles, revealing significant differences in both the number and the type of phytochemicals with significantly high (high level) and low (low level) peak areas between cowpea varieties in each growing season (2020 and 2021) (Figure 2a,b). All data points are directly visualized on the heatmap as color squares. The color spectrum demonstrates whether values are high (red) or low (blue). Phytochemical peak areas exhibited significant variability among varieties and seasons, with most compounds showing increased peak areas during the 2020 growing season compared to 2021. In each variety, the number of metabolites that showed the highest (red color intensity) and lowest (blue color intensity) peak areas are as follows: WP (27; 8), CPPH (9;11), CL (6;4), QA (3;10), MS (4;14) and CAB (3;17) in 2020 (Figure 2a), and QA (17;5), CL (9;1), WP (4;8), CPPH (4;14), MS (2;14) and CAB (0:22), in 2021 (Figure 2b).

In 2020, the WP variety had the richest metabolite profile, with 27 metabolites with the highest peak areas. These metabolites include phytohormones (such as jasmonic acid); alkaloids (such as reserpine); phenolic compounds, including phenolic acids and flavonoids (such as gallic acid, gentisic acid, ortho-hydroxyphenylacetic acid, 4-hydroxyphenylpyruvic acid, and catechol); fatty acid derivatives (such as corchorifatty acid F and azelaic acid); amino acids (such as proline); and other defense-related compounds (Figure 2a). This variety was followed by the QA variety in 2021, with 17 phytochemicals exhibiting the highest peak areas, including important signaling molecules and other stress-related metabolites such as salicylic acid, oleamide, fumaric acid, apigenin, dihydrotestosterone, alpha-linolenoyl ethanolamide, and ethyl tetradecanoate (Figure 2b). CPPH in 2020 and CL in 2021 showed moderate metabolite abundances (nine metabolites each) in their respective highest peak areas across both years, with metabolites including aesculin, xanthine, octadecanamide, palmitic amide, linoleoyl ethanolamide, isoleucine, linoleic acid, leucine, and L-phenylalanine in CPPH and alpha-linolenic acid, 3-cresotinic acid, leucine, isoleucine, homogentisic acid, phenylacetaldehyde, 4-hydroxybenzaldehyde, trans-cinnamic acid, and phenol in the CL variety. In 2020, these compounds also included 4-hydroxybenzaldehyde, trans-cinnamic acid, ferulic acid, oleanolic acid, carvone, and cuminaldehyde in CL. The remaining varieties exhibited low metabolic profiles in the highest peak area (from two to four metabolites); MS in 2020 (glycitein, daidzin, homogentisic acid, and adenosine), WP in 2021 (phytochemicals; 2-piperididone, kaempferol, kynurenic acid, and palmitic acid), and CPPH in 2021 (vindoline, jasmonic acid, aesculin, and ergocalciferol) each had four phytochemicals. QA (alpha-linolenic acid, palmitic acid, and apigenin) and CAB (guanine, ethyl tetradecanoate, and salicylic acid) both had three phytochemicals in 2020, and MS had two in 2021, including guanine and carvone (Figure 2a,b).

The phytochemicals with the highest peak areas exclusively detected in 2020 include key phenolics and stress-related metabolites such as gentisic acid, reserpine, thymine, gallic acid, catechol, azelaic acid, suberic acid, 16-hydroxy hexadecanoic acid, ortho-hydroxyphenylacetic acid, 4-hydroxyphenylpyruvic acid, corchorifatty acid F, and 2-piperidinone, which were highest in the WP variety, followed by CPPH (with increased levels of L-phenylalanine, isoleucine, and palmitic amide), CL (cuminaldehyde, ferulic and oleanolic acid) and MS (glycitein), This suggests strong season-specific activation of defense and/or antioxidant pathways (Figure 2a).

In 2021, the phytochemicals with a higher peak area than in 2020 include vindoline and ergocalciferol (in CPPH); phenylacetaldehyde and phenol (CL); trans-cinnamic acid (in both CL and MS); kaempferol (WP); and palmitic acid (in the WP variety, followed by CL). Although palmitic acid was highly expressed in 2021, it was also observed in 2020 (in QA and CAB), but not at a higher level than that observed in WP in 2021 (Figure 2a,b). This fatty acid was also detected using GC-MS as a FAME (methyl palmitate) and was also high in QA (2020) and CL (2021) (Figure 1h). Jasmonic acid increased more in CPPH (2021) than in WP (2020); homogentisic acid was highly increased in CL (2021) compared to MS (2020) and decreased in WP and CPPH in both seasons. These phytochemicals are linked to specialized metabolism and secondary defense.

Other phytochemicals had the highest peak areas in both 2020 and 2021, either within the same or different varieties. Aesculin was highly expressed in CPPH in both seasons. Salicylic acid was highest in QA (2021), followed by CAB and CPPH in both seasons, with a large increase in 2020. Kynurenic acid had the highest peak area in WP in both seasons, which was higher in 2020, followed by QA; the lowest peak areas were observed in CAB (2020), CL (2021), and CPPH and MS (in both seasons). Alpha-linolenic acid had the highest peak area in QA (2020) and the lowest peak area in WP and MS in 2021 (Figure 2a,b).

Kaempferol was the highest in WP and the lowest in CPPH, while adenosine increased in MS and QA but was lowest in CAB. Phenylacetaldehyde was the highest in MS, CL, and WP and the lowest in CAB and CPPH (Figure 2a,b), indicating that this compound is variety-dependent regardless of the season.

The CAB variety had the highest number (17 and 22) of phytochemicals with the lowest peak area in 2020 and 2021, respectively (Figure 2a,b). The phytochemicals isoliquiritigenin, glycitein, ferulic acid, (Z)-3-methyl-2-(2-pentenyl)-2-cyclopenten-1-one, and benzoic acid decreased in the CAB variety in both seasons. D-malic acid and trans-cinnamic acid peak areas were high in CL and MS in both seasons and were lowest in the CAB and QA varieties in 2021 and in CPPH in both seasons.

These patterns suggest that the conditions in 2020 triggered a wider and stronger metabolite diversity compared to 2021; however, distinct metabolites were observed.

**Figure 3:**
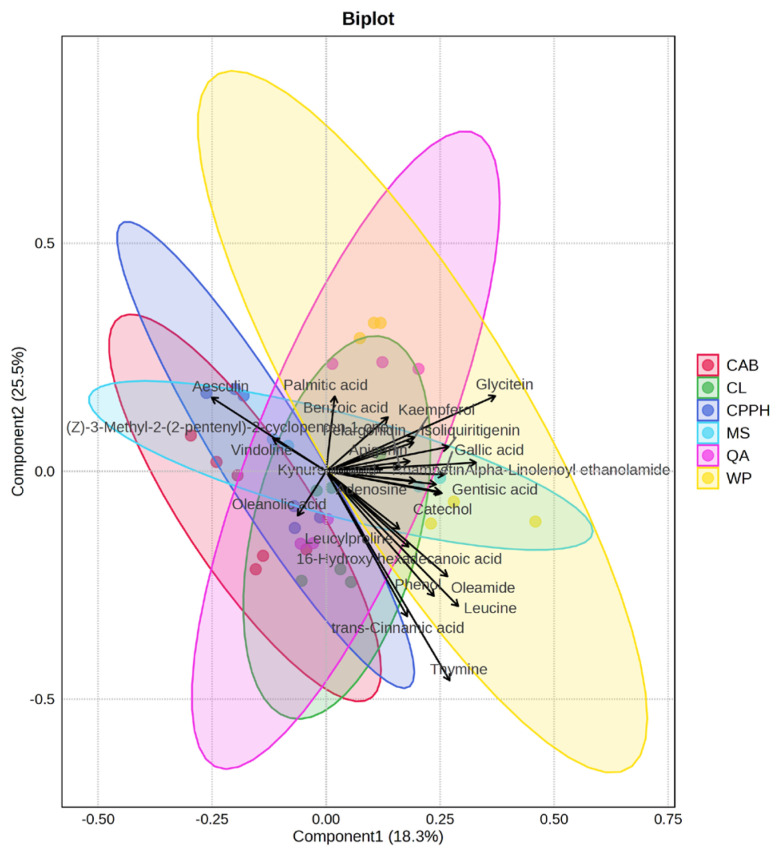
Partial least squares discriminant analysis (PLS-DA) biplot of metabolic profiles across six cowpea varieties, showing a clear discrimination between groups (CAB, CL, CPPH, MS, QA, and WP cowpea varieties) based on the phytochemical data. The colored ellipses represent 95% confidence intervals for each group. Loading vectors indicate the contribution of individual metabolites to group separation. Each point represents an individual sample, and the vector length indicates the magnitude of metabolite contribution to the discriminant model. Note: CAB—California Blackeye Pea, CL—Colossus, CPPH—Coronet Pinkeye Purple Hull, MS—Mississippi Silver, QA—Queen Anne, and WP—Whippoorwill Steele’s Black.

The partial least squares discriminant analysis (PLS-DA) (Figure 3) discriminated against six varieties of cowpea using metabolic profiling, with a total variance of 43.8%. Components 1 (18.3%) and 2 (25.5%), with distinct cowpea-specific clustering patterns and minimal confidence ellipse overlap, showed distinct separation for most cowpea varieties, confirming statistically significant metabolic differences. The WP variety exhibited the most extensive distribution along the axis of positive component 1, indicating the highest metabolic variability and a distinct metabolic profile. The fact that this variety exhibits the highest metabolic variability and a wide distribution suggests a robust adaptive capacity and enhanced metabolic resilience under varying weather conditions. CAB and QA showed pronounced separation along component 2, with CAB positioned in the upper-left quadrant and QA extending toward the lower region. The CL, CPPH, and MS varieties displayed intermediate positioning; CPPH exhibited a compact, well-defined cluster in the upper-left region, with minimal overlap with other groups, while CL and MS showed some degree of overlap, suggesting more similar metabolic profiles. After WP, CPPH demonstrated the second highest-performing group due to its metabolic consistency and clear separation, while MS represented the lowest-performing group due to its significant overlap with other groups (including CL and CPPH) and the least distinctive metabolomic profile, with few unique discriminating metabolites.

Key discriminating metabolites include aesculin, glycitein, gallic acid, palmitic acid, alpha-linolenoyl ethanolamide, and adenosine, representing comprehensive primary and secondary metabolic reprogramming underlying the observed differences under different weather conditions, suggesting biosynthesis takes place through different pathways and not through a single pathway.

**Figure 4:**
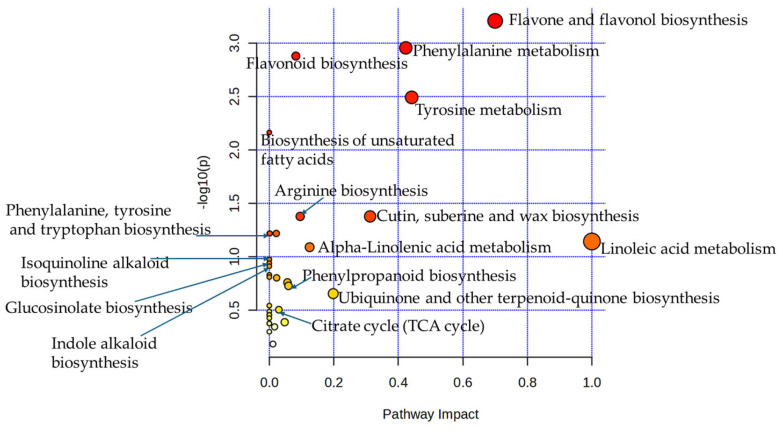
Metabolic pathway analysis in response to environmental stress. Red dots represent the most enriched pathways, and yellow dots represent less significantly enriched pathways.

In the metabolic pathway enrichment analysis, a total of 34 metabolic pathways were revealed to have been reprogrammed in response to heat stress. Five metabolic pathways showed significantly altered regulation (with raw *p* < 0.05, FDR < 0.10), with a particular emphasis on secondary metabolite biosynthesis, aromatic acid metabolism, fatty acid biosynthesis, and membrane structural maintenance (Figure 4, Table A2 in Appendix A). Flavone and flavonol biosynthesis was most significantly altered (*p* = 0.00062, FDR = 0.040, and the high biological impact of 0.7), followed by phenylalanine metabolism (*p* = 0.001, FDR = 0.04), flavonoid biosynthesis (*p* = 0.001, FDR = 0.04), tyrosine metabolism (*p* = 0.003, FDR = 0.07), and unsaturated fatty acid biosynthesis (*p* = 0.007, FDR = 0.126). Phenylalanine and tyrosine metabolism, indicating coordinated disruption of aromatic amino acid catabolism, was characterized by perturbation of other pathways, including flavonoid, flavone, and flavonol biosynthesis, indicating multiple secondary metabolite biosynthesis pathways were disturbed. Cutin, suberine, and wax biosynthesis, along with arginine biosynthesis, had a significant raw *p*-value of 0.042; however, their FDR was 0.55, indicating a non-significant difference. Additionally, the biological impacts were moderate (0.31) for cutin, suberine, and wax biosynthesis and low (0.1) for arginine biosynthesis. Also, alpha-linolenic acid metabolism had a low biological impact (0.125) and was not significantly different between seasons (*p* = 0.081, FDR = 0.623) (Figure 4 and Table A2 in the Appendix A).

Linoleic acid metabolism also exhibited the maximum biological impact (impact score of 1.0) despite lacking statistical significance (*p* = 0.07 and FDR = 0.601) (Figure 4, Table A2), indicating that a few altered metabolites occupy topologically critical positions within the metabolic network. This pattern suggests targeted modifications of specific regulatory nodes or rate-limiting enzymes, potentially generating substantial biological effects without extensive pathway perturbation. Disruptions in linoleic acid production suggest the induced compensatory increases in alternative fatty acids, demonstrating how changes in metabolically central compounds influence broader lipid homeostasis. The absence of statistical significance likely reflects precise metabolic regulation rather than pathway dysfunction, suggesting a sophisticated strategy where cowpeas make targeted adjustments to critical control points while maintaining cellular function and optimizing resource allocation under stress conditions.

The biosynthesis of unsaturated fatty acid pathway had an impact score of zero, despite significant enrichment (Table A2), due to the methodological distinction between pathway enrichment analysis and topological impact assessment. Impact scores are weighted according to the topological importance and centrally affected metabolites within the biochemical network, while statistical significance indicates a greater-than-expected number of altered metabolites within the pathway.

These observed metabolic profiles show that cowpeas respond to heat stress by producing antioxidants, adjusting their membranes, and shifting their metabolism to maintain cell stability during environmental challenges.

## 3. Discussion

We were unable to identify studies that have directly examined the impact of weather conditions on the composition of fatty acids/FAMEs in cowpeas grown in the field. However, some studies have reported metabolites associated with herbivorous insects and pathogens as playing insecticidal and antimicrobial roles and having drought effects in a greenhouse environment. Therefore, our comparisons are based on the findings of other legumes related to cowpeas and other crops. Additionally, no studies were found referencing the specific cowpea varieties used in our research.

In 2020, there was a significant increase in the concentration of methyl laurate, methyl palmitate, elaidic oleic c + t, behenate, and lignocerate, with the highest concentrations observed in the QA variety. This could be attributed to the elevated temperature and rainfall in this season. Rahnama et al. reported similar findings in sunflower (*Helianthus annuus*), where high temperatures (30–32 °C during the day and 24–25 °C at night) led to a significant increase in palmitic and stearic acids [48]. On the other hand, a temperature between 21/13 °C and 37/29 °C (day/night) significantly altered the fatty acid composition in soybean through an increase in the concentration of oleic acid and a decrease in the concentrations of linoleic and linolenic acids, while stearic and palmitic acids showed significant interaction between cultivars [49]. In contrast, Kurasiak-Popowska et al. found that in *Camelina sativa*, temperatures ranging from 14 to 23 °C had no significant effect on the composition of the studied fatty acids (oleic, linoleic, linolenic, eicosenoic, and erucic acids) [50].

The same compounds that exhibited an increased concentration in 2020 decreased in 2021, which could be attributed to a decrease in temperature, which directly affects plant secondary metabolites. These climate parameters are known to influence the synthesis of secondary metabolites, with such responses varying by plant species and variety. The synthesis of plant metabolites is a hereditary trait, influenced by environmental conditions that can affect the composition and quantity of fatty acids [48] and other phytochemicals.

Temperature-responsive fatty acid regulation is a fundamental adaptive mechanism that enables plants to maintain cellular homeostasis and survive thermal fluctuations in the natural environment [51]. To sustain membrane fluidity and integrity, plants dynamically adjust their lipid composition; in particular, they decrease their lipid unsaturation in response to elevated temperatures [52,53] and increase their membrane saturation levels [54]. The proportions of unsaturated and saturated fatty acids in membrane lipids are fundamental factors in thermostability and temperature acclimation [55]. In our study, we observed an increase in the concentration of saturated fatty acids, including methyl laurate, methyl palmitate, behenate, and lignocerate, which could have contributed to enhanced membrane bilayer integrity and cuticular barriers during stress. In contrast, monounsaturated oleic acid accumulated due to a reduced desaturase activity, balancing rigidity and flexibility. Similarly, Falcone et al. showed that there is regulated membrane unsaturation under elevated growth temperatures in Arabidopsis, with increases in saturated palmitic acid (16:0), diunsaturated linoleic acid (18:2), and monounsaturated oleic acid (18.1) and a decrease in unsaturated fatty acids 16:3 and 18:3 [55]. This variation could be attributed to the growth stage, the temperature range, and mixed pathway activation. This adaptive response is regulated by temperature-dependent regulation of fatty acid desaturase enzymes [56].

Further, the high impact of linoleic acid metabolism supports the biological significance of this regulation, showing that linoleic acid occupies a central role in the fatty acid metabolic network [57]. It coordinates and regulates the production of saturated fatty acids, thereby helping to maintain membrane integrity under thermal stress. Moreover, linoleic acid serves as a precursor for oxylipin signaling molecules such as jasmonic acid, which regulate the gene expression that in turn modulates heat stress responses and antioxidant defenses important for preserving membrane integrity under stress [54]. In addition, under heat stress, the alteration of peripheral fatty acids is prioritized over central enzymatic nodes in cowpea, as shown by the zero-impact score of the biosynthetic unsaturated fatty acid pathway. This strategy optimizes membrane fluidity while minimizing disruption to key metabolic pathways. By adjusting fatty acid synthesis, cowpea demonstrates adaptive allocation of resources to prioritize immediate stress defenses while preserving/maintaining regular fatty acid biosynthesis. The contrasting roles of the unsaturated fatty acid biosynthesis pathway and linoleic acid metabolism suggest an intricate regulatory mechanism. The interplay between saturated and unsaturated fatty acids in cowpeas under heat stress remains complex and not fully understood. Further studies integrating lipidomics, transcriptomics, and enzymology will help to clarify these mechanisms and provide deeper insights into plant heat stress adaptation strategies.

LC-MS/MS analysis revealed that the cowpea variety, growing season, and their interactive effects significantly influenced phytochemical biosynthesis. Phytochemicals exhibited variety-dependent patterns, with the increases in peak areas in some cowpea varieties regardless of seasonal conditions, for instance, in kaempferol and adenosine levels, indicating genetic control over their synthesis and accumulation (Figure 2a,b; Table A1). This aligns with the findings of Mageney et al., who analyzed 28 kale cultivars and observed significant differences in the content of kaempferol glycoside and in quercetin-to-kaempferol ratios, indicating genetic diversity in flavonoid biosynthesis pathways [58]. Bang et al. reported that both genotype and harvest year influenced kaempferol levels across *Amaranthus* species [59], suggesting both genetic and environmental influences on phytochemical accumulation [59,60]. Variations in climatic conditions affect the types of phytochemicals observed in plants and their amount [60]. Weather conditions had a more pronounced effect on the levels of some phytochemicals, such as jasmonic acid, uracil, proline, and cuminaldehyde, than the variety (Table A1). Our findings demonstrated that the combined effects of elevated temperature and increased rainfall in 2020 corresponded with increased phytochemical peak areas. This finding aligns with previous studies showing that increased temperature enhanced secondary metabolite production, though the role of rainfall appears more complex. Moreira et al. reported that increased temperatures increased the community-weighted mean of total phenolics in annual plants, while reduced rainfall had no effect [61], indicating that weather conditions, particularly temperature, influence phytochemical synthesis in plants. In contrast, our study observed that elevated temperatures combined with increased rainfall led to enhanced accumulation of metabolites in cowpeas, suggesting that phytochemical responses vary with different levels of environmental stress. While temperature is a main driver of metabolite biosynthesis, rainfall modulates this effect. This suggests that the optimal combination of environmental variables, rather than a single factor, significantly influences the phytochemical profiles of plants. Furthermore, the interactive effects between temperature and rainfall warrant consideration.

Environmental factors, particularly temperature, have been shown to significantly affect phytochemical contents in crops such as wheat and strawberries in other studies [62,63]. Shamloo et al. observed that growing wheat at higher temperatures (up to 30 °C) led to increased levels of total phenolic acids, flavonoids, and palmitic and oleic acids, while linoleic and linolenic acids were reduced, a likely defensive response to heat stress [64].

Temperature’s effect on secondary metabolites is species-specific. For instance, in the grass species *Paspalum wettsteinii*, flavonoids, proline, and organic acids were upregulated at high temperatures (35 °C, 40 °C, and 45 °C), concurrent with an induction of heat shock proteins through the activation of stress response genes and a downregulation of free fatty acids [65]. In *Brassicaceae*, temperature stress due to increased temperatures reduced carotenoid and beta-carotene levels [66], increased accumulation of terpene in *Daucus carota* in response to heat shock [44], decreased salicylic acid and phenolic contents in *Populus tremula* [67], and increased catechin levels in tea plants [44]. Selinga et al. determined which heat-induced proteins were associated with cowpea performance and photosynthesis recovery under high temperatures in a controlled environment (40 °C for 72 h) and field conditions (during summer at two South African research sites—a warm site with a maximum temperature of 26–28 °C and a hot site with a maximum temperature of 30–32 °C). They observed a higher regulation of heat shock proteins and succinic dehydrogenase at the hot site [68]. In our study, flavonoids (glycitein) and phenolic acids (gentisic acid) were increased at high temperatures, while salycilic acid, phenolic acids (homogentisic acid), and alkaloids (vindoline) were increased at low temperatures.

The upregulation and downregulation of metabolites depend on the genes responsible for their biosynthesis and their tolerance to temperature [66]. Different varieties showed specific responses in relation to the effect of weather, with an increase in L-phenylalanine and palmitic amide in the CPPH variety and cuminaldehyde in the CL variety, with a decrease in these phytochemicals in the CAB variety, during the high-temperature 2020 season. Our findings aligned with those of Goufo et al., who studied cowpea metabolites in two varieties (Pinhel and Fradel) under drought stress in a greenhouse setting with day/night temperatures of approximately 43.6/19.0 °C. They found that proline, quercetin 3-O-6″-malonyglycoside, galactinol, quercetin, and phenylalanine were highly increased in the leaves, and kaempferol 3-O-diglycoside and quercetin 3-O-6″-malonyglycoside were highly increased in the roots of the Pinhel variety in comparison to Fradel [25]. Plant metabolites employ dual defense strategies through up- and downregulation mechanisms. Upregulation activates defensive genes to combat stress, pathogens, and environmental challenges, while downregulation conserves energy for essential processes such as photosynthesis and reproduction [69]. The increased temperature and rainfall observed in 2020 could have created favorable conditions for insect pest proliferation and pathogen establishment, which would subsequently trigger plant regulatory gene networks governing stress responses. This environmental stress induced different metabolic defense strategies; upregulation of diverse secondary metabolites, including alkaloids (reserpine), flavonoids (catechol), and phenolic acids (gallic acid) in the WP variety, provided active defense against stresses. Conversely, the downregulation of primary metabolites such as fumaric acid and adenosine likely contributed to a strategic trade-off mechanism to conserve metabolic energy while maintaining essential processes as an alternative defense during prolonged heat stress conditions.

Although metabolites were detected in leaves, other studies suggest that other plant parts, including seeds, can accumulate similar compounds under these conditions. Shamloo et al. reported an increase in the accumulation of phytochemicals in seeds grown under heat stress [64]. Mayer et al. exposed cowpea cells to heat shock at 42 °C and observed an increase in proline, leucine, and isoleucine after 24 h [70]. These amino acids were similarly increased in our study in 2020, where maximum daily temperatures reached 32 °C. However, unlike our findings, phenylalanine was found to be downregulated in Mayer’s study.

This study demonstrates that seasonal (environmental) and varietal (genotypic) differences significantly influence the production and accumulation of primary and secondary metabolites in cowpea, with genotypic traits determining how heat-induced signals are transduced into metabolite biosynthesis responses through transcription factor expression, pathway sensitivity, and precursor availability [71]. The biosynthetic pathways identified in cowpeas were grouped into four primary mechanisms in response to weather conditions: aromatic amino acid response, secondary metabolite defense systems, membrane lipid regulation, and integrated stress response mechanisms. The aromatic amino acid response was mediated by phenylalanine and tyrosine metabolism. The former was significantly activated by three key metabolites (L-phenylalanine, phenylacetaldehyde, and ortho-hydroxyphenyl acetic acid), providing carbon skeletons for phenylpropanoid pathways and facilitating the synthesis of various defensive compounds [72], including flavonoids, phenolics, and benzenoid/phenylpropanoids that mitigate ultraviolet radiation [73] and heat stress. Phenylalanine is an important amino acid for protein synthesis and a precursor to other metabolites and secondary metabolites [73]. The substantial increase in these phytochemical levels in 2020 may have triggered cascading biosynthetic pathways, resulting in increased production of secondary metabolites, including flavonoids, alkaloids, and phenolics, as demonstrated by their elevated peak areas. Tyrosine is an important amino acid for protein biosynthesis; it can also be involved in the biosynthesis of secondary metabolites in plants [74]. Tyrosine metabolism was altered through metabolites such as homogentisic acid, 4-hydroxyphenylpyruvic acid, and fumaric acid, which are also crucial for the synthesis of other secondary metabolites, including tyrosine-derived phytochemicals such as suberin and tocopherols, which are structural phytochemicals for plant protection [73].

Homogentisic acid levels were highest in CL (2021), followed by MS (2020). As a phenolic compound involved in tyrosine metabolism, homogentisic acid contributed to metabolite formation during heat stress in both seasons and served defensive functions. It acts as a precursor for lipid-soluble antioxidants, such as tocopherol, which protect photosynthetic membranes during abiotic stress [75]. The secondary metabolite defense system was primarily controlled through the following significantly identified pathways: flavone, flavonol, and flavonoid biosynthesis. This led to the production of antioxidant compounds that function as reactive oxygen species scavengers, including apigenin, pelargonidin, isoliquiritigenin, and kaempferol, maintaining membrane stability and regulating pathways that respond to heat stress [76,77,78]. Similar to our findings, flavonoid biosynthesis was reported to reduce oxidative damage from heat and salt stress in tomatoes [76]. In addition, transgenic rice plants accumulate kaempferol under stress conditions, reducing oxidative damage and cell death, suggesting that flavonoid synthesis enhances the tolerance to combined heat and salt stress through multiple physiological and molecular mechanisms [79]. These flavonoids regulate heat shock factors and proteins while modulating gene expression through hormone cross-signaling molecules such as salicylic acid and abscisic acid [79,80].

Membrane lipid regulation was achieved through the biosynthesis of fatty acids, linoleic acid metabolism, and the biosynthesis of cutin, suberine, and wax, triggering the synthesis of structural materials for maintaining cell wall integrity. Cutin, suberine, and wax are important structural materials for this purpose, evidenced by the presence of suberic acid in leaves and plant roots during stress, aiding protection [81]. Fatty acids are precursors for the synthesis of secondary metabolites and maintain optimal membrane fluidity to ensure proper cellular function during stress conditions [55,82].

The integrated stress response involved the redirection of resources from the primary metabolism to secondary metabolite production, with increased amino acid synthesis linked to the synthesis of heat shock proteins. A similar finding was presented in a previous study in rice, in which flavone accumulation activated heat shock proteins and improved heat tolerance [79]. These interconnected pathways work synergistically to provide comprehensive tolerance to heat stress in cowpea varieties, suggesting conserved mechanisms between plant species that warrant further investigation, particularly regarding secondary metabolite, amino acid, and fatty acid metabolism and their relationship with heat stress responses.

## 4. Materials and Methods

### 4.1. Research Site

The field study was conducted on a certified organic research farm located at the George Washington Carver Agricultural Experiment Station, Tuskegee University, Alabama, USA, with the following GPS coordinates: latitude: 32°26′10.976″ N, longitude: −85°44′11.307″ W. Extraction was conducted in the chemistry department at Tuskegee University, while LC-MS/MS and GC/MS analyses were conducted at Auburn University.

### 4.2. Climatic Conditions

Climatological parameters, including precipitation, relative humidity, and temperature during the summers of 2020 and 2021, were obtained from the National Water and Climate Center (NWCC), USDA–Natural Resources Conservation Service, from the Tuskegee University Weather Station SCAN site (Tuskegee (2115)—Site Information and Reports (usda.gov) (https://wcc.sc.egov.usda.gov/nwcc/site?sitenum=2115, accessed on 26 February 2024). Weather data were analyzed using analysis of variance in OriginPro 2025 software. Mean separation was performed using Tukey’s test with a significance threshold of *p* < 0.05.

### 4.3. Variety Selection and Experiment Design

Organic certified seeds of six cowpea varieties—Mississippi Silver (MS), Colossus (CL), California Black Eye Pea (CAB), Coronet Pinkeye Purple Hull (CPPH), Queen Anne (QA), and Whippoorwill Steele’s Black (WP)—were obtained from the Southern Exposure Seed Exchange (Mineral, VA, USA) and Garden Hoard (Howell, MI, USA). In each season, the experimental design was a Latin square with six blocks, with a total area of 1547.76 square meters. Each block was treated as a replicate with six subplots (each with a cowpea variety), and each subplot had three rows; the middle row was for the experiment, and two were guard rows. Each row was 6.10 m long. There was a 3.05 m separation between blocks, a 3.05 m separation between subplots, and a 1.22 m separation between rows; cowpea seeds were planted 8 inches (0.20 m) apart within rows. Organic farming practices were observed, which included certified organic seeds, crop rotation, mechanical weeding, and no spraying of synthetic chemicals. Cowpeas were grown in and exposed to natural conditions.

### 4.4. Sample Collection and Preparation

Six weeks after planting (during the flowering stage), leaves were collected randomly from the six cowpea varieties in the middle row and stored in separate freezable Ziploc bags in a freezer at −80 °C (So-Low Environmental Equipment, Cincinnati, OH, USA) until use. Samples from both seasons were collected at comparable stages. The samples were air-dried and ground to a powder form using a heavy grinder, followed by further storage at 4 °C until use.

### 4.5. Extraction of FAMEs

FAMEs were extracted following the method of Abugri et al. and Abugri and Pritchett [83,84], with little modification. Extraction was carried out under a fume hood: a methanol and HCl mixture in a 4:1 ratio *v*/*v* was prepared in conical flasks. A total of 2 mL of the methanol and HCl mixture was added to 0.5 g of dry, ground leaf tissue in 16 × 100 mm PYREX screw-cap culture tubes and vortexed for 1 min. The tubes were placed in dry heating blocks at 100 °C for 30 min and cooled on ice for 5 min, after which 2 mL of hexane was added, followed by 1 mL of distilled water. This mixture was vortexed and then centrifuged at 2000 rpm for 5 min. The top layer containing the FAMEs was transferred to gas liquid chromatography (GLC) vials using Pasteur pipettes, stored at −20 °C, and later analyzed by GC-MS at Auburn University. Extraction was repeated three times.

#### GC-MS Analysis

An Agilent 6890N GC and 5975 MS (Agilent Technologies, Santa Clara, CA, USA) with a Restek Stabilwax-DA column (30 m × 250 mm, 0.25 µm) were used to analyze the FAMEs. The temperature of the injection port and MS transfer line was set at 250 °C at a constant pressure of 22 psi. The temperature of the GC oven was programmed as follows: initial temperature of 50 °C for 2.5 min, ramp to 180 °C at 50 °C/min, ramp to 212 °C at 3 °C/min, and ramp to 250 °C at 50 °C/min with a hold of 3 min. Thus, a total run time of 33 min was required. Raw data was processed and analyzed using Agilent enhanced ChemStation E.02.02.1431. A FAME standard (Supelco 37 Component FAME Mix (Supelco, Bellefonte, PA, USA) was used to quantify the concentration in the extracts with the use of a methyl nonadecanoate internal standard at a concentration of 50 ug/mL. The concentrations of FAME Mix ranged from 350 ug/mL to 1 ug/mL. The retention times of the standard FAME Mix were compared to those in the NIST 2.0 spectral library to identify the standard and sample chromatographic peaks by unique spectral features. Calibration curves were employed to quantify sample concentrations, with detailed calibration parameters provided in the Appendix A (Table A3). The limit of detection (LOD) and limit of quantification (LOQ) were estimated from the lowest level calibration point with signal-to-noise ratios of 3 and 9, respectively. The results were reported in terms of the concentration of the identified fatty acids in each cowpea variety based on the concentrations of the FAME Mix in ug/mL. The two-way analysis of variance (ANOVA) was performed using Prism GraphPad 10 software to assess mean variations in FAME concentrations between cowpea varieties and growing seasons.

### 4.6. Extraction of Other Phytochemicals

A total of 0.5 g of ground leaves was placed in each reaction tube, and 10 mL of methanol was added to each reaction tube. The mixture was vortexed for 15 s, followed by maceration at room temperature for 48 h in the dark. The extract was first filtered through filter paper, and the same amount of solvent previously added was added to the plant material. The extraction was repeated until the filtrate was clear. The filtrate was further filtered into rotavapor vials through a 0.22 µm syringe filter and then stored at 4 °C. The solvent was removed from the extract using a vacuum evaporator; Rotavapor R-215 (BUCHI Corporation, Meierseggstrasse, Flawil, Switzerland; BUCHI Corporation New Castle, DE, USA) at 150 rpm and 40 °C. The dry crude extract was stored at room temperature in the dark until further use in LC-MS/MS analysis.

#### Characterization of Extracts Using LC-MS/MS Analysis

Methanol extract analysis was carried out on a Vanquish ultra-high performance liquid chromatography (UHPLC) system (Thermo Fisher, Waltham， MA, USA) coupled with a quadrupole Orbitrap mass spectrometer (Orbitrap Exploris 120, Thermo) with electrospray ionization (H-ESI) in positive and negative mode using Xcalibur software (V4.4.16.14). A total of 10 µL of the sample was injected onto a C18 column (ACQUITY UPLC^®^ BEH C18, 1.7 µm, 2.1  ×  50 mm, Waters, Milford, MA, USA) maintained at 40 °C with a 200 μL/min flow rate of mobile phase solution A (0.1% formic acid in water) and mobile phase B (95% acetonitrile, 4.9% water, and 0.1% formic acid) beginning at 0% B, held for 1 min, increased to 2% B at 7 min, increased to 28% B at 9 min, increased to 38% B at 11 min, increased to 49% B at 13 min, increased to 70% B at 15 min, increased to 89% B at 17 min, then increased to 100% B at 19 min held for 1 min, followed by re-equilibration to 0% B for 8 min for a total analysis time of 28 min. The samples were chilled to 10 °C while the column was heated to 40 °C. The MS scan range was 70–1000 *m*/*z* with a resolution of 60,000, a standard AGC target, a 70% RF lens, and an auto maximum injection time, with EASY-IC on. The spray voltage was 3500 V in positive mode and 2500 V in negative mode, the ion transfer tube temperature was 320 °C, the vaporizer temperature was 275 °C, and the sheath and aux gases were 45 and 5, respectively. There was nontargeted fragmentation with exclusion of compounds found in the blank injections. The isolation window was 2, the collision energy was normalized to 30%, and the Orbitrap resolution was 15,000, with other parameters set to auto. The instrument used is incapable of determining stereochemistry, and positional isomers are likely a reflection of the compounds in the databases rather than discrimination in the actual data. Compounds were named by matching fragment ions to mzCloud and/or chemical formula using the Compound Discoverer 3.2.0.421 Software. Data were screened via both manual searches and enrichment analysis. The compounds with the best match (mzCloud Best Match > 70%) were selected and subjected to MetaboAnalyst 6.0 software, for the enrichment analysis to cross-validate the compound names matched against Human Metabolome (HMDB), PubChem, and Kyoto Encyclopedia of Genes and Genomes (KEGG) databases to confirm compound identification. The peak area data of selected compounds were analyzed using MetaboAnalyst 6.0, which included a two-way ANOVA with multiple testing correction for false discovery rate (FDR) based on the adjusted *p*-value (q-value) < 0.05; heatmaps were constructed to compare the phytochemical differences among varieties and growing seasons, and a PLS-DA was performed to discriminate between varieties and determine the metabolites contributing most to their separation. Additional information for compound identification, including calculated molecular weights (Calc. MW), chemical formulas, and retention times (RTs), is included in the ANOVA table (Table A1) in the Appendix A.

Pathway analysis for metabolite biosynthesis was performed using MetaboAnalyst 6.0 software with reference to the KEGG pathway in the *Arabidopsis thaliana* database; metabolites showing significant enrichment (both raw *p* < 0.05 and FDR < 0.10) and/or with biological impact scores were explained.

## 5. Conclusions

The prolonged high temperatures in the summer of 2020 induced significant heat stress in cowpeas, which triggered comprehensive metabolic reprogramming, characterized by increased FAME production and enhanced secondary metabolite diversity. These metabolic alterations support the hypothesis that environmental conditions drive differences in the amounts and compositions of defensive phytochemicals between growing seasons. Varietal responses exhibited distinct patterns: the QA variety exhibited an increased concentration of FAMEs, while the WP variety displayed diverse phytochemical profiles with a high peak intensity associated with stress regulation. The identified phytochemicals play crucial roles in plant defense mechanisms, enabling adaptation to environmental challenges and protection against abiotic and biotic stressors. These findings have significant implications for crop improvement strategies, including the selection of the variety and planting time. The identified stress-responsive phytochemicals represent valuable genetic resources for developing heat-tolerant cowpea varieties capable of withstanding pest pressure and disease susceptibility under adverse climatic conditions. Future research should focus on elucidating the enzymology and molecular mechanisms underlying heat tolerance in selected varieties and characterizing temperature-dependent metabolic pathway regulation in different growth stages. Understanding these stress-responsive metabolic networks is essential for optimizing breeding programs and developing cowpea-derived biopesticides for integrated pest management strategies, ultimately contributing to enhanced agricultural resilience under changing environmental conditions.

## Figures and Tables

**Table 1:** Average (mean ± SE) values for relative humidity, rainfall, and temperature for the study years during the summer season (2020 and 2021) and during individual months (for temperature) within the season. Means followed by the same letter are not statistically significant. Max = maximum temperature, min = minimum temperature, SE = standard error, and CV = coefficient of variation.

	Season				June		July		August	
	Relative Humidity (%)	Rainfall (mm)	Max (°C)	Min (°C)	Max (°C)	Min (°C)	Max (°C)	Min (°C)	Max (°C)	Min (°C)
2020	97.92 ± 0.54 ^a^	1423 ± 13.19 ^a^	32.02 ± 0.21 ^a^	20.21 ± 0.19 ^a^	30.98 ± 0.33 ^a^	18.88 ± 0.40 ^a^	32.58 ± 0.33 ^a^	21.01 ± 0.18 ^a^	32.47 ± 0.36 ^a^	20.7 ± 0.21 ^a^
2021	97.66 ± 0.57 ^a^	774 ± 3.17 ^b^	29.88 ± 0.23 ^b^	18.80 ± 0.21 ^b^	29.50 ± 0.33 ^b^	17.91 ± 0.32 ^a^	28.84 ± 0.37 ^b^	18.14 ± 0.17 ^b^	31.29 ± 0.38 ^b^	20.32 ± 0.39 ^a^
*p* value	0.74	<0.0001	<0.0001	<0.0001	0.0027	0.0617	<0.0001	<0.0001	0.0279	0.3952
CV (%)	0.055	0.084	0.069	0.097	0.06	0.11	0.064	0.049	0.065	0.085

## Data Availability

Data are contained within the article.

## Abbreviations

The following abbreviations are used in this manuscript:

AL	Alabama
ANOVA	Analysis of variance
CAB	California Black Eye Pea
CAENS	College of Agriculture, Environment and Nutrition Sciences
CL	Colossus
CPPH	Coronet Pinkeye Purple Hull
GC-MS	Gas chromatography–mass spectrometry
GWCAES	George Washington Carver Agricultural Experiment Station
FAMEs	Fatty acid methyl esters
FDR	False discovery rate
HCl	Hydrochloric acid
HMDB	Human Metabolome Database
KEGG	Kyoto Encyclopedia of Genes and Genomes
LC-MS/MS	Liquid chromatography–tandem mass spectrometry
LOD	Limit of detection
LOQ	Limit of quantification
MS	Mississippi Silver
NIFA	National Institute of Food and Agriculture
NIST	National Institute of Standards and Technology
NWCC	National Water and Climate Center
QA	Queen Anne
RT	Retention time
UFA	Unsaturated fatty acids
USA	United States of America
USDA	United States Department of Agriculture
WP	Whippoorwill Steele’s Black

## Appendix A

**Table A1:** Analysis of variance showing the 62 metabolites significantly affected by cowpea variety, season, and their interaction, with additional compound information, including calculated molecular weight (Calc. MW), chemical formula, and retention time (RT).

Phytochemical Name	Formula	Calc. MW	RT (min)	Cowpea Variety (F.val)	Cowpea Variety (raw.p)	Cowpea Variety (adj.p)	Seasons (F.val)	Seasons (raw.p)	Seasons (adj.p)	Interaction (F.val)	Interaction (raw.p)	Interaction (adj.p)
Glycitein	C16 H12 O5	284.06849	13.9	202.82	8.19 × 10^−19^	5.57 × 10^−17^	78.146	5.16 × 10^−9^	1.95 × 10^−8^	35.918	2.20 × 10^−10^	2.14 × 10^−9^
Rhamnetin	C16 H12 O7	316.05832	13.907	177.8	3.81 × 10^−18^	1.30 × 10^−16^	31.796	8.33 × 10^−6^	2.098 × 10^−5^	8.119	0.000134	0.0003375
Aesculin	C15 H16 O9	340.07931	11.688	147.1	3.44 × 10^−17^	7.80 × 10^−16^	53.3	1.53 × 10^−7^	4.34 × 10^−7^	3.46	0.017	0.02513
D-Malic acid	C4 H6 O5	134.02148	12.879	103.13	2.04 × 10^−15^	3.47 × 10^−14^	76.56	6.24 × 10^−9^	2.23 × 10^−8^	7.397	0.000254	0.0005956
Phenol	C6 H6 O	94.04183	12.141	89.653	1.00 × 10^−14^	1.36 × 10^−13^	66.681	2.19 × 10^−8^	6.77 × 10^−8^	8.472	0.0000988	0.0002799
Argininosuccinic acid	C10 H18 N4 O6	290.11873	13.23	70.806	1.42 × 10^−13^	1.61 × 10^−12^	26.152	3.12 × 10^−5^	7.072 × 10^−5^	2.25	0.082	0.10521
Salicylic acid	C7 H6 O3	138.03166	7.727	69.137	1.85 × 10^−13^	1.80 × 10^−12^	196.22	4.77 × 10^−13^	3.60 × 10^−12^	49.927	6.62 × 10^−12^	1.13 × 10^−10^
Chrysin	C15 H10 O4	254.05787	13.759	58.774	1.12 × 10^−12^	9.52 × 10^−12^	9.597	5.00 × 10^−3^	0.0077273	140.59	5.81 × 10^−17^	3.95 × 10^−15^
Isoliquiritigenin	C15 H12 O4	256.07359	15.295	55.159	2.24 × 10^−12^	1.69 × 10^−11^	0.313	0.581	0.61731	38.816	9.75 × 10^−11^	1.33 × 10^−9^
Ferulic acid	C10 H10 O4	194.05786	12.225	50.908	5.36 × 10^−12^	3.64 × 10^−11^	8.653	7.00 × 10^−3^	0.010348	6.817	0.000437	0.0009905
Homogentisic acid	C8 H8 O4	168.04218	11.777	45.384	1.85 × 10^−11^	1.14 × 10^−10^	87.106	1.86 × 10^−9^	7.91 × 10^−9^	65.33	3.48 × 10^−13^	7.89 × 10^−12^
Gentisic acid	C7 H6 O4	154.0265	3.746	40.64	6.00 × 10^−11^	3.40 × 10^−10^	18.921	2.17 × 10^−4^	0.0004307	13.656	0.0000023	1.20 × 10^−5^
trans-Cinnamic acid	C9 H8 O2	148.05239	12.143	39.938	7.22 × 10^−11^	3.78 × 10^−10^	92.105	1.09 × 10^−9^	4.94 × 10^−9^	8.157	0.000129	0.0003374
Catechol	C6 H6 O2	110.03672	3.743	36.419	1.90 × 10^−10^	9.23 × 10^−10^	18.839	2.22 × 10^−4^	0.0004307	8.784	0.0000761	0.000225
Daidzin	C21 H20 O9	416.11089	12.66	35.555	2.44 × 10^−10^	1.11 × 10^−9^	657.23	5.99 × 10^−19^	2.04 × 10^−17^	100.9	2.62 × 10^−15^	8.91 × 10^−14^
Pelargonidin	C15 H10 O5	270.05284	13.917	31.263	9.21 × 10^−10^	3.91 × 10^−9^	7.368	1.20 × 10^−2^	0.017362	36.371	1.93 × 10^−10^	2.14 × 10^−9^
Gallic acid	C7 H6 O5	170.02144	1.565	24.816	9.34 × 10^−9^	3.74 × 10^−8^	5.946	2.30 × 10^−2^	0.030667	9.613	0.0000389	0.000147
L-Phenylalanine	C9 H11 N O2	165.07891	3.2	24.006	1.29 × 10^−8^	4.87 × 10^−8^	519.8	8.99 × 10^−18^	1.53 × 10^−16^	15.923	6.03 × 10^−7^	4.10 × 10^−6^
Vindoline	C25 H32 N2 O6	456.23372	12.799	21.547	3.68 × 10^−8^	1.32 × 10^−7^	12.038	2.00 × 10^−3^	0.0032381	8.889	0.0000698	0.000225
Benzoic acid	C7 H6 O2	122.03674	13.151	20.144	6.97 × 10^−8^	2.37 × 10^−7^	26.147	3.12 × 10^−5^	7.072 × 10^−5^	8.808	0.0000746	0.000225
3-Cresotinic acid	C8 H8 O3	152.04728	12.353	15.991	5.8 × 10^−7^	1.86 × 10^−6^	956.78	7.52 × 10^−21^	5.11 × 10^−19^	28.004	2.81 × 10^−9^	2.39 × 10^−8^
(Z)-3-Methyl-2-(2-pentenyl)-2-cyclopenten-1-one	C11 H16 O	164.12007	15.306	15.881	6.17 × 10^−7^	1.86 × 10^−6^	7.091	1.40 × 10^−2^	0.019833	11.029	0.0000134	0.0000536
Apigenin	C15 H10 O5	270.05276	14.624	15.798	6.47 × 10^−7^	1.86 × 10^−6^	4.475	0.045	0.056667	7.972	0.000152	0.0003691
Xanthine	C5 H4 N4 O2	152.03339	12.848	15.716	6.77 × 10^−7^	1.86 × 10^−6^	145.43	1.13 × 10^−11^	7.68 × 10^−11^	6.027	0.00095	0.0019
Kynurenic acid	C10 H7 N O3	189.04263	11.745	15.698	6.84 × 10^−7^	1.86 × 10^−6^	4.642	0.041	0.052604	3.697	0.013	0.020091
16-Hydroxy hexadecanoic acid	C16 H32 O3	272.23477	20.512	11.744	8.09 × 10^−6^	2.12 × 10^−5^	396.62	1.97 × 10^−16^	2.23 × 10^−15^	11.425	0.0000101	4.29 × 10^−5^
4-Hydroxybenzaldehyde	C7 H6 O2	122.03674	11.66	10.872	0.000015	3.78 × 10^−5^	20.85	1.25 × 10^−4^	0.0002576	4.18	0.007	0.012205
Oleamide	C18 H35 N O	264.24532	20.914	9.035	0.0000618	0.0001501	567.83	3.25 × 10^−18^	7.37 × 10^−17^	15.399	8.12 × 10^−7^	4.68 × 10^−6^
Ortho-Hydroxyphenylacetic acid	C8 H8 O3	152.04727	6.521	7.021	0.00036	0.0008441	18.746	2.28 × 10^−4^	0.0004307	6.133	0.000854	0.0018148
Isoleucine	C6 H13 N O2	131.09454	1.422	6.968	0.000379	0.0008591	390.96	2.32 × 10^−16^	2.25 × 10^−15^	15.368	8.26 × 10^−7^	4.68 × 10^−6^
Carvone	C10 H14 O	150.1044	13.204	6.843	0.000426	0.0009345	0.834	0.37	0.41246	3.378	0.019	0.026917
Oleanolic acid	C30 H48 O3	478.34286	20.361	6.293	0.000728	0.0015228	5.933	2.30 × 10^−2^	0.030667	3.201	0.024	0.03264
Isoquinoline	C9 H7 N	129.05771	12.915	6.278	0.000739	0.0015228	0.06	0.808	0.83248	11.878	7.37 × 10^−6^	3.34 × 10^−5^
Linoleic acid	C18 H32 O2	280.24024	21.057	6.135	0.000852	0.0016592	104.7	3.13 × 10^−10^	1.64 × 10^−9^	3.685	0.013	0.020091
Suberic acid	C8 H14 O4	174.08913	12.759	6.132	0.000854	0.0016592	215.73	1.71 × 10^−13^	1.45 × 10^−12^	9.083	0.0000595	0.0002023
Fumaric acid	C4 H4 O4	116.01098	1.224	5.798	0.001	0.0018889	22.762	7.44 × 10^−5^	0.0001581	4.96	0.003	0.0055135
Azelaic acid	C9 H16 O4	188.10468	13.011	5.238	0.002	0.0035789	79.984	4.15 × 10^−9^	1.66 × 10^−8^	5.142	0.002	0.0038857
Ergocalciferol	C28 H44 O	396.33939	21.735	5.127	0.002	0.0035789	1.006	0.326	0.37803	4.195	0.007	0.012205
Guanine	C5 H5 N5 O	151.04937	1.531	4.802	0.003	0.0049756	38.346	2.13 × 10^−6^	5.57 × 10^−6^	13.025	3.44 × 10^−6^	1.67 × 10^−5^
Linoleoyl ethanolamide	C20 H37 N O2	323.28241	19.639	4.974	0.003	0.0049756	9.227	6.00 × 10^−3^	0.0090667	1.938	0.125	0.15455
Palmitic acid	C16 H32 O2	273.26681	16.566	4.803	0.003	0.0049756	16.219	4.92 × 10^−4^	0.0009042	5.055	0.003	0.0055135
2-Piperidinone	C5 H9 N O	99.06835	2.77	4.691	0.004	0.0064762	73.488	9.09 × 10^−9^	3.09 × 10^−8^	1.802	0.151	0.18336
Ethyl tetradecanoate	C16 H32 O2	256.24015	20.074	4.117	0.008	0.012651	13.493	1.00 × 10^−3^	0.0017	8.399	0.000105	0.0002856
Kaempferol	C15 H10 O6	286.0478	12.374	3.905	0.01	0.015455	0.006	0.937	0.937	1.004	0.437	0.45024
Adenosine	C10 H13 N5 O4	267.09683	1.398	3.852	0.011	0.016622	3.834	0.062	0.076655	1.654	0.184	0.21951
Leucine	C6 H13 N O2	131.09457	1.629	3.497	0.016	0.022667	135.1	2.41 × 10^−11^	1.49 × 10^−10^	3.808	0.011	0.01781
Thymine	C5 H6 N2 O2	126.04288	1.463	3.493	0.016	0.022667	452.79	4.37 × 10^−17^	5.94 × 10^−16^	6.027	0.00095	0.0019
Alpha-Linolenic acid	C18 H30 O2	278.22457	20.324	3.494	0.016	0.022667	23.617	5.93 × 10^−5^	0.0001301	2.325	0.074	0.096769
Phenylacetaldehyde	C8 H8 O	120.05746	12.563	3.269	0.022	0.030531	0.999	0.328	0.37803	1.257	0.314	0.33362
Uracil	C4 H4 N2 O2	112.02722	1.248	2.715	0.044	0.05984	13.479	0.001	0.0017	2.092	0.101	0.12719
Neocnidilide	C12 H18 O2	176.12005	15.839	2.658	0.048	0.064	28.482	1.78 × 10^−5^	4.32 × 10^−5^	9.197	0.0000542	0.000194
Palmitic amide	C16 H33 N O	255.25625	20.705	2.621	0.05	0.065385	112.62	1.52 × 10^−10^	8.61 × 10^−10^	3.398	0.018	0.026043
Corchorifatty acid F	C18 H32 O5	328.22417	14.584	2.463	0.062	0.079547	15.385	6.41 × 10^−4^	1.15 × 10^−3^	3.563	0.015	0.022667
Dihydrotestosterone	C19 H30 O2	290.22462	19.365	2.356	0.071	0.089407	11.999	2.00 × 10^−3^	3.24 × 10^−3^	17.225	2.97 × 10^−7^	2.24 × 10^−6^
Reserpine	C33 H40 N2 O9	608.26352	19.8	2.323	0.074	0.091491	102.14	3.99 × 10^−10^	1.94 × 10^−9^	3.167	0.025	0.033333
Alpha-Linolenoyl ethanolamide	C20 H35 N O2	321.26674	17.68	2.278	0.079	0.095929	1.237	0.277	0.33046	6.264	0.000749	0.001643
Leucylproline	C11 H20 N2 O3	228.14737	11.6	1.988	0.117	0.13717	42.96	8.86 × 10^−7^	2.41 × 10^−6^	3.344	0.02	0.027755
Octadecanamide	C18 H37 N O	283.28756	22.018	1.931	0.126	0.14522	69.375	1.53 × 10^−8^	4.95 × 10^−8^	3.865	0.01	0.016585
Proline	C5 H9 N O2	115.06327	1.276	1.743	0.163	0.1817	55.436	1.10 × 10^−7^	3.25 × 10^−7^	1.142	0.366	0.38289
Cuminaldehyde	C10 H12 O	148.08875	12.364	1.247	0.318	0.34324	6.478	0.018	0.02498	0.898	0.498	0.498
4-Hydroxyphenylpyruvic acid	C9 H8 O4	180.04219	13.663	0.956	0.464	0.493	9.535	0.005	0.0077273	3.933	0.01	0.016585
Jasmonic acid	C12 H18 O3	210.12554	14.863	0.923	0.483	0.50529	4.805	0.038	0.049692	0.963	0.46	0.46687

**Table A2:** Pathway analysis showing the most affected pathways in metabolite biosynthesis in response to stress.

Pathway	Total	Expected	Hits	Raw p	−Log10(p)	Holm Adjust	FDR	Impact
Flavone and flavonol biosynthesis	10	0.18457	3	0.00062	3.2073	0.057085	0.040597	0.7
Phenylalanine metabolism	12	0.22149	3	0.00111	2.9548	0.10098	0.040597	0.42308
Flavonoid biosynthesis	47	0.8675	5	0.001324	2.8782	0.11914	0.040597	0.08216
Tyrosine metabolism	17	0.31378	3	0.003223	2.4917	0.28687	0.074136	0.44134
Biosynthesis of unsaturated fatty acids	22	0.40606	3	0.006861	2.1636	0.60378	0.12625	0
Arginine biosynthesis	18	0.33223	2	0.041895	1.3778	1	0.55062	0.09568
Cutin, suberine, and wax biosynthesis	18	0.33223	2	0.041895	1.3778	1	0.55062	0.3125
Valine, leucine, and isoleucine biosynthesis	22	0.40606	2	0.060457	1.2186	1	0.5562	0
Phenylalanine, tyrosine, and tryptophan biosynthesis	22	0.40606	2	0.060457	1.2186	1	0.5562	0.0015
Alanine, aspartate, and glutamate metabolism	22	0.40606	2	0.060457	1.2186	1	0.5562	0.02159
Linoleic acid metabolism	4	0.07383	1	0.07188	1.1434	1	0.60118	1
alpha-Linolenic acid metabolism	26	0.47989	2	0.081315	1.0898	1	0.62341	0.125
Isoquinoline alkaloid biosynthesis	6	0.11074	1	0.10593	0.975	1	0.74963	0
Glucosinolate biosynthesis	65	1.1997	3	0.11486	0.93982	1	0.75129	0
Indole alkaloid biosynthesis	7	0.1292	1	0.12249	0.91189	1	0.75129	0
Valine, leucine, and isoleucine degradation	37	0.68293	2	0.14739	0.83154	1	0.80423	0
Tropane, piperidine, and pyridine alkaloid biosynthesis	9	0.16612	1	0.15475	0.81038	1	0.80423	0
Purine metabolism	75	1.3843	3	0.15735	0.80313	1	0.80423	0.02272
Pyrimidine metabolism	41	0.75676	2	0.17362	0.76041	1	0.84067	0.05624
Phenylpropanoid biosynthesis	43	0.79367	2	0.18702	0.72811	1	0.86031	0.05935
Ubiquinone and other terpenoid-quinone biosynthesis	48	0.88596	2	0.22116	0.6553	1	0.96888	0.19809
beta-Alanine metabolism	18	0.33223	1	0.28627	0.54322	1	1	0
Citrate cycle (TCA cycle)	20	0.36915	1	0.31269	0.50488	1	1	0.0295
One carbon pool by folate	21	0.38761	1	0.32555	0.48738	1	1	0
Fatty acid elongation	23	0.42452	1	0.35057	0.45523	1	1	0
Pyruvate metabolism	23	0.42452	1	0.35057	0.45523	1	1	0
Pantothenate and CoA biosynthesis	25	0.46144	1	0.37469	0.42633	1	1	0
Glycosylphosphatidylinositol (GPI)-anchor biosynthesis	28	0.51681	1	0.40926	0.388	1	1	0.04762
Cyanoamino acid metabolism	29	0.53527	1	0.42037	0.37637	1	1	0
Biosynthesis of various plant secondary metabolites	29	0.53527	1	0.42037	0.37637	1	1	0
Arginine and proline metabolism	32	0.59064	1	0.4525	0.34438	1	1	0.01637
Fatty acid degradation	37	0.68293	1	0.50227	0.29906	1	1	0
Fatty acid biosynthesis	56	1.0336	1	0.65453	0.18407	1	1	0.01123

**Table A3:** GC-MS FAME calibration curve information.

Compound	Target Ion and Qualifier Ions	RT Min	Quadratic Term	Linear Term	Constant Term	Correlation Coefficient (r^2^)
10:0 methyl decanoate	74.2, 87.2, 55.15, 186.4	5.2	1.561e−005	3.815e−003	1.598e−003	0.999
12:0 methyl laurate	74.2, 87.15, 55.15, 214.40	5.8	1.548e−005	4.004e−003	3.019e−003	0.999
14:0 methyl myristate	74.2, 87.15, 55.15, 242.4	6.7	1.780e−005	3.890e−003	3.610e−003	0.999
Methyl palmitate	74.2, 87.2, 55.15, 270.6	8.1	2.918e−005	5.769e−003	5.368e−003	0.999
16:1 methyl palmitoleate	55.15, 74.2, 69.15, 268.4	8.3	2.638e−006	7.876e−004	−3.739e−004	0.999
18:0 Methyl stearate	74.2, 7.2, 55.15, 98.4	10.3	0.000e+000	4.689e−003	−2.116e−003	0.998
18:1 elaidic oleic cis & trans	55.15, 74.2, 69.15, 296.4	10.55	1.296e−005	2.069e−003	1.766e−003	0.999
18:2n−6 linolelaidate	65.15, 55.15, 81.2, 294.4	14.0	5.593e−006	1.618e−003	−5.778e−004	0.999
18:3n−6 linolenate	67.5, 74.2, 55.15, 150	14.2	0.000e+000	2.673e−005	3.958e−004	0.574
18:3n−3 linolenate	67.15, 79.15. 55.15, 292.4	14.7	1.000e−006	6.153e−004	−1.126e−003	0.998
22:0 behenate	74.15, 87.2, 55.2, 354.6	20.4	1.741e−005	3.296e−003	−9.790e−004	0.999
24:0 lignocerate	74.2, 87.2, 55.2, 382.4	22.9	2.426e−005	2.509e−003	−1.856e−003	0.999
